# Learning Moisture‐Induced Damage From Vision: Diffusion Models for Real‐Time Monitoring of Additive Manufacturing Processes

**DOI:** 10.1002/advs.76062

**Published:** 2026-07-01

**Authors:** Jiyoung Jung, Yuna Yoo, Dharneedar Ravichandran, Dahyun Daniel Lim, Grace X. Gu

**Affiliations:** ^1^ Department of Mechanical Engineering University of California Berkeley California USA; ^2^ School of Mechanical Engineering Korea University Seoul Republic of Korea

**Keywords:** additive manufacturing, diffusion model, moisture effect, real‐time monitoring

## Abstract

Moisture is a subtle but critical factor in polymer manufacturing, particularly in additive manufacturing (AM) processes. Many polymers, including thermoplastic polyurethane, are highly hygroscopic and readily absorb ambient moisture, which can lead to defects such as stringing, pores, and bubbles. These defects degrade both print quality and mechanical performance, posing a significant challenge for AM as a reliable next‐generation manufacturing technology. Due to these challenges, real‐time monitoring and integrity estimation of fabricated parts have become essential to ensure manufacturing quality control. Here, we create an in situ visual monitoring system for fused filament fabrication using an optical camera‐based setup to detect moisture‐induced degradation and evaluate the quality of printed parts. A diffusion model‐based anomaly detection framework is devised to identify the degradation. Our model can identify filaments affected by moisture and assess the extent of degradation from captured images. Furthermore, the system demonstrates that the detected anomaly score is closely correlated with the mechanical performance of the printed parts, offering a nondestructive evaluation approach. These results show that an integrated visual monitoring system and generative artificial intelligence models can provide a robust foundation for enhancing the reliability of additive manufacturing and support resource‐efficient sustainability through early, nondestructive detection of defects.

## Graphical abstract

We introduce a vision‐based real‐time monitoring system for additive manufacturing that detects subtle moisture‐induced degradation via a diffusion model‐based framework. The approach enables nondestructive assessment of moisture‐induced damage level and mechanical performance and establishes a practical route toward more intelligent, reliable, and sustainable manufacturing processes.

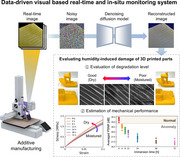

## 1 Introduction

Moisture is a quiet but consequential variable in polymer manufacturing. Many polymers absorb moisture from ambient air, and even small amounts can trigger plasticization and property loss [1, 2, 3]. In melt‐based processes, absorbed moisture can vaporize during heating and create bubbles, pores, and unstable flow. These challenges are significant when manufacturing relies on consistent rheology and controlled deposition. The issue becomes even more pronounced in additive manufacturing (AM), where materials are processed locally, incrementally, and at elevated temperatures [4, 5]. Among AM processes, material extrusion, particularly fused filament fabrication (FFF), has become the most widely adopted due to its cost‐effectiveness, accessibility, and versatility [6, 7, 8, 9]. FFF also offers a practical route to directly fabricate soft and compliant structures using low‐modulus thermoplastic elastomers (TPEs), a rapidly growing area of interest. Thermoplastic polyurethane (TPU), the most widely used TPE in FFF, is increasingly leveraged across industries because of its high elasticity, abrasion resistance, and low melting temperature [10]. TPU is additionally attractive as a more sustainable alternative to conventional elastomers due to its reprocessability and durability [11]. Despite these advantages, a critical drawback of TPU is that it is highly hygroscopic, readily absorbing ambient moisture from the surrounding environment [12], which induces physical degradation of the polymer through plasticization [3]. During printing, this moisture can vaporize inside the heated nozzle, causing defects such as stringing, pores, bubbles, and nonuniform flow, creating discontinuities [12, 13]. Such defects severely degrade the surface finish and mechanical integrity of printed parts, thereby hindering consistent quality assurance in industrial production and posing a major challenge to reliable and high‐quality manufacturing.

To address these moisture‐induced issues, conventional methods such as predrying filaments [13] or using desiccant‐based storage [14] have been explored. However, the effectiveness of desiccant‐based solutions diminishes over time and with repeated container opening. Furthermore, oven drying before each print is impractical for manufacturing productivity and efficiency and still fails to fully eliminate residual moisture, as evidenced by persistent surface bubbling [12]. Even with these pre‐treatments, from a manufacturing industry perspective, implementing real‐time monitoring systems and predictive models remains essential for ensuring the consistent quality and performance of fabricated parts. Previous research on FFF printing has primarily focused on investigating the general effects of humidity on the prints, revealing the mechanism of moisture‐induced degradation [3, 12, 15]. Specifically, these studies have demonstrated that moisture absorption leads to the formation of structural voids and a significant decrease in stiffness and ultimate strength. In parallel, studies on real‐time monitoring have been largely limited to the visual detection of under/over‐extrusion using cameras and machine learning (ML) models [16, 17, 18, 19]. This is because such defects exhibit a direct and clear correlation with surface morphology, whereas the effect of moisture‐induced degradation remains inherently ambiguous under visual inspection. Therefore, the previous studies on quantifying moisture content or degradation levels have primarily relied on destructive and offline characterization methods, such as thermogravimetric analysis (TGA) [20] or post hoc microscopic inspection of internal damage [21]. Due to these diagnostic hurdles, real‐time in situ monitoring of humidity‐induced damage remains largely unaddressed. However, developing such techniques is essential for immediate anomaly detection and process optimization, ensuring manufacturing efficiency and reliability. Another major challenge is predicting the mechanical properties of printed parts using visual data alone. Such a nondestructive approach is essential for increasing productivity and ensuring product reliability. Ultimately, despite its importance, the real‐time assessment of moisture‐induced degradation and its impact on mechanical properties through visual monitoring remains unexplored.

In this study, we create a data‐driven framework for real‐time, in situ monitoring of the FFF printing process. Our system enables anomaly detection that distinguishes between dry and moisture‐affected prints, evaluates the extent and severity of moisture impact, and nondestructively estimates the mechanical property degradation based on a strong correlation between the stiffness of printed parts and anomaly score. A major challenge in implementing data‐driven artificial intelligence (AI) methodologies in manufacturing is the severe data imbalance, where anomaly data is significantly scarcer than normal data. To overcome this limitation, we have adopted a diffusion model‐based approach trained exclusively on normal (dry) condition data. Diffusion models are generative models that learn to reconstruct data by reversing a gradual noise‐addition process, allowing them to identify anomalies by measuring reconstruction errors [22, 23]. Beyond identifying anomalies, our model leverages reconstruction errors to estimate moisture‐driven degradation, thereby establishing a direct link between visual data and degradation level. Finally, to connect process observations to performance, we experimentally establish a correlation map between moisture absorption level and the resulting mechanical property (Young's modulus). This map enables the system to directly correlate Young's modulus with in situ visual monitoring data through the estimated damage severity. While identifying subtle moisture‐induced degradation levels has traditionally necessitated posthoc microscopy, our AI‐driven framework enables real‐time and nondestructive monitoring using only a cost‐effective camera. From a sustainability perspective, this system serves as a technical cornerstone for resource‐efficient manufacturing by mitigating both environmental and economic costs. By enabling the immediate detection of moisture‐induced defects, the framework prevents unnecessary material waste and the substantial energy expenditure associated with reprinting failed components. Furthermore, by establishing a nondestructive evaluation approach that correlates visual data with mechanical integrity, it eliminates the need for resource‐intensive destructive testing that typically renders manufactured parts unusable. This transition from reactive, posthoc characterization to proactive in situ monitoring minimizes the carbon footprint and time‐consuming trial‐and‐error cycles. Consequently, by enabling real‐time anomaly detection and property assessment, this work provides a practical step toward a smart manufacturing system that not only ensures high‐quality control and real‐time process optimization but also facilitates the integration of sensitive materials into a sustainable manufacturing ecosystem with minimal waste.

## 2 Results and Discussions

**FIGURE 1:**
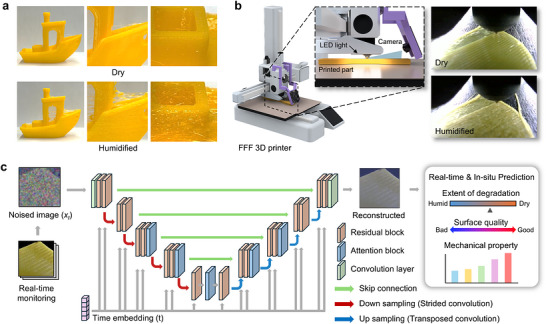
(a) Effect of moisture absorption on 3D‐printed TPU specimens. Images of the 3DBenchy printed with dry and humidified (immersed in water for 24 h) TPU filaments are compared. (b) Experimental setup of the real‐time, in situ monitoring system for FFF printing. The system consists of a camera module and an LED lighting module integrated into the FFF 3D printer. (c) Overall framework of the diffusion model‐based framework for evaluating moisture‐induced damage. The framework takes printing images as input to estimate the extent of damage, which is directly related to surface quality, and subsequently correlates these damage levels with the degraded mechanical performance of the manufactured parts.

TPU is a hygroscopic polymer, and absorbed moisture can severely degrade the surface quality of printed parts, as shown in Movie S1 and Figure S1. To demonstrate this effect, Figure 1a compares 3DBenchy printed with dry and humidified filaments (immersed in water for 24 h). While the part printed with dry filament exhibits a smooth surface and minimal stringing, the one from humidified filament shows a highly irregular surface and extensive stringing. To assess the extent of degradation in the printed part, this study aims to develop a real‐time monitoring system for parts fabricated via FFF 3D printing. To monitor the printed surface during the printing process, a commercial USB camera and a light source were installed on the print head of the Bambu FFF printer, as shown in Figure 1b. To prioritize manufacturing productivity, images were captured during the printing process without pausing the print head, albeit at the cost of image resolution. A digital photograph of the monitoring setup is presented in Figure S2. By leveraging a diffusion model‐based framework, our system evaluates the extent of moisture‐induced degradation and the resulting mechanical performance loss of the printed parts from the real‐time visual monitoring of the printing process, as depicted in Figure 1c.

### 2.1 Effect of Moisture Absorption on TPU 3D Printing

**FIGURE 2:**
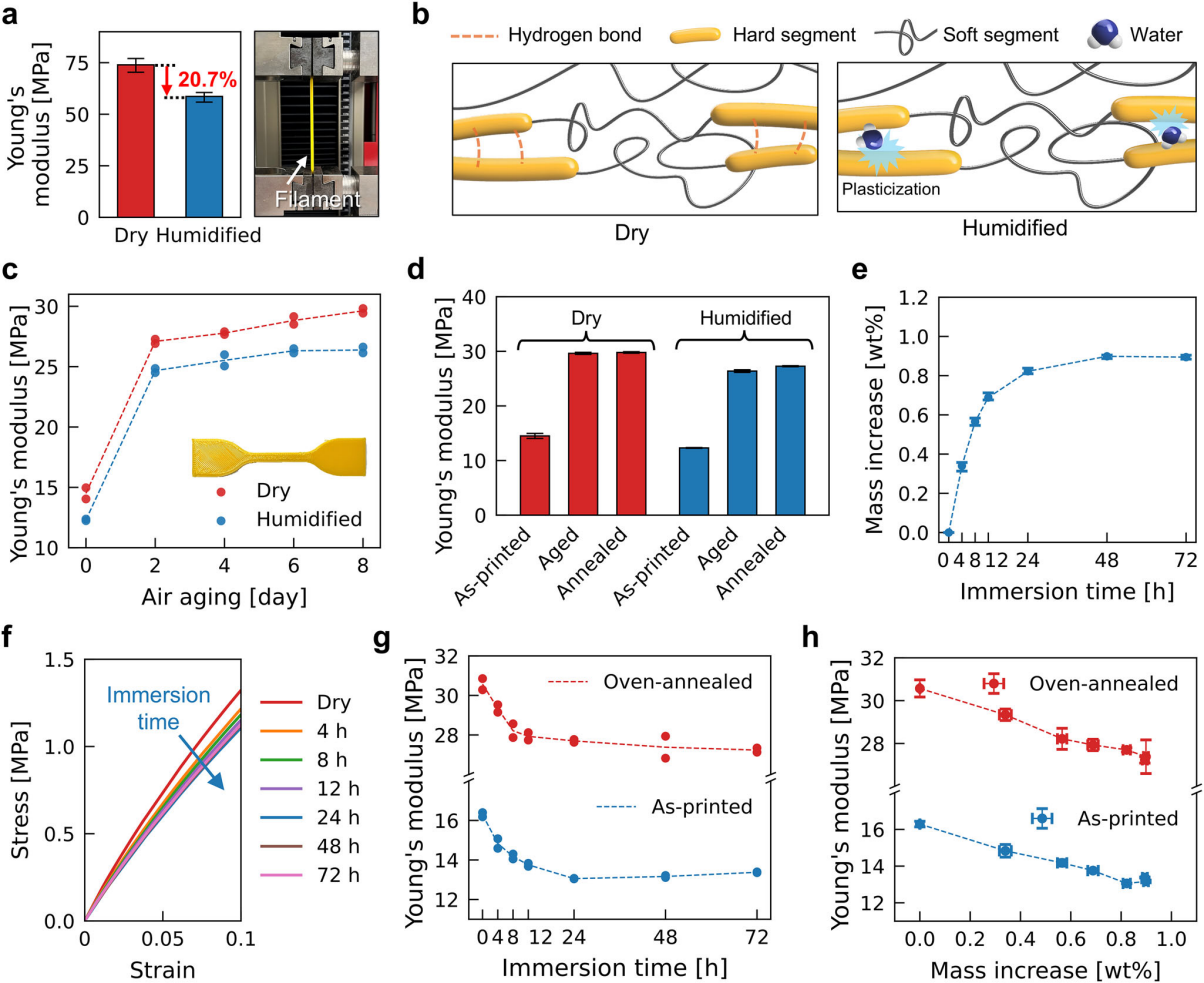
Comprehensive analysis of mechanical property changes in TPU due to moisture absorption. (a) Stiffness variation in TPU filaments according to moisture absorption (dry vs. 24‐h water immersion) (*n*  =  3; error bars indicate minimum and maximum values). (b) Schematics of plasticization in the TPU polymer chain induced by water molecules. (c) Changes in Young's modulus of 3D‐printed ASTM specimens as a function of aging duration at room temperature under ambient conditions (*n*  =   2). (d) Comparison of Young's modulus for specimens under different postprocessing states: as‐printed, aging in the air for 8 days, and thermal annealing in an oven for 4 h (*n*  =  2). (e) Mass increase (wt.%) of the filament with immersion time (*n*  =  3). (f) Stress‐strain curves of as‐printed specimens using filaments with various water immersion times. (g) Comparison of Young's modulus as a function of filament immersion time for as‐printed and oven‐annealed specimens (*n*  =  2). (h) Correlation between Young's modulus and mass increase, derived from (e) and (g).

As a preliminary step toward constructing the proposed real‐time monitoring system, the fundamental impact of moisture on 3D printing with TPU was thoroughly investigated. First, the effects of moisture absorption on the filament property were evaluated. Two sets of specimens were prepared: pristine (dry) filaments and filaments immersed in water for 24 h (humidified). Tensile tests were conducted on these two sets of filaments to characterize their mechanical behavior, as shown in Figure 2a. The humidified filaments show a 20.7% reduction in Young's modulus compared to dry filaments, indicating that moisture absorption significantly degrades the mechanical performance of the polymeric material. This is consistent with previous studies [24] showing that absorbed moisture in TPU acts as a plasticizer, weakening secondary links between polymer chains as illustrated in Figure 2b and consequently reducing the mechanical properties.

To understand the variations in the mechanical properties of printed parts under various operational conditions, we investigated the post‐printing hardening phenomenon. Figure 2c illustrates the evolution of mechanical properties for specimens printed from dry and humidified (immersed in water for 24 h) filaments when exposed to ambient air. In both cases, a hardening phenomenon is observed during air aging, characterized by a rapid initial increase in stiffness that subsequently decelerates and approaches a plateau over time. This hardening phenomenon is believed to be primarily caused by the gradual reorganization of polymer chains and the increased density of hydrogen‐bonded hard segments [25, 26]. This aging effect in ambient air can be accelerated by applying heat to the printed specimens. Figure 2d compares the mechanical properties of dry and humidified filament specimens under three conditions: as‐printed, air‐aged for 8 days, and oven‐annealed for 4 h. It is of note that the oven‐annealed specimens exhibited characteristics similar to those aged in air for 8 days. Compared to the as‐printed specimens, the oven‐annealed and air‐aged specimens showed 104.2% and 105.4% higher stiffness for dry filaments, and 114.4% and 121.6% higher for humidified filaments, respectively. Overall, this shows that 4 h of oven annealing can be used to replicate the mechanical properties achieved after 8 days of air aging, allowing for rapid evaluation of postprinting hardening behavior.

Finally, the effect of water immersion time on the printed parts was investigated. In this study, immersion time was employed as a metric to represent moisture‐induced degradation, with longer immersion durations representing prolonged exposure to a humid environment, greater moisture uptake, and correspondingly higher levels of damage. To validate this approach, the mass increase (wt.%) of filaments was investigated as a function of immersion time. As shown in Figure 2e, the mass increased rapidly during the first 24 h, attributed to the fast initial diffusion of water molecules into the polymer matrix. The water uptake reached saturation at approximately 0.894 wt.% as the immersion time approached 72 h, suggesting that the diffusion process approached equilibrium. To examine the mechanical degradation of the printed parts associated with water uptake in the filaments, the mechanical properties of the printed parts were evaluated both immediately after printing (as‐printed) and after 4 h of oven annealing, which was used in place of long‐term air aging to accelerate the experiments. The stress‐strain (S‐S) curves of the specimens as a function of immersion time are presented in Figure 2f. It is observed that longer exposure to moisture leads to a greater degradation of mechanical performance. The evolution of Young's modulus for as‐printed and oven‐annealed specimens is investigated as a function of immersion time in Figure 2g. For both types of specimens, Young's modulus exhibits a sharp decline during the early stages of moisture exposure, followed by a plateau after approximately one day of exposure. This rapid initial reduction corresponds well with the rapid increase in moisture absorption observed in the filament during the first 24 h. Figure 2h presents Young's modulus as a function of mass increase by combining data from Figure 2e,g. The results reveal a linear relationship between moisture content in filament and Young's modulus, indicating that moisture absorption is closely correlated with the mechanical properties of the printed parts. Moisture absorption led to a decrease in mechanical properties of up to 19.8% for as‐printed specimens and 10.9% for oven‐annealed specimens compared to dry conditions, which highlights the necessity of a monitoring system to ensure reliable, high‐quality manufacturing.

**FIGURE 3:**
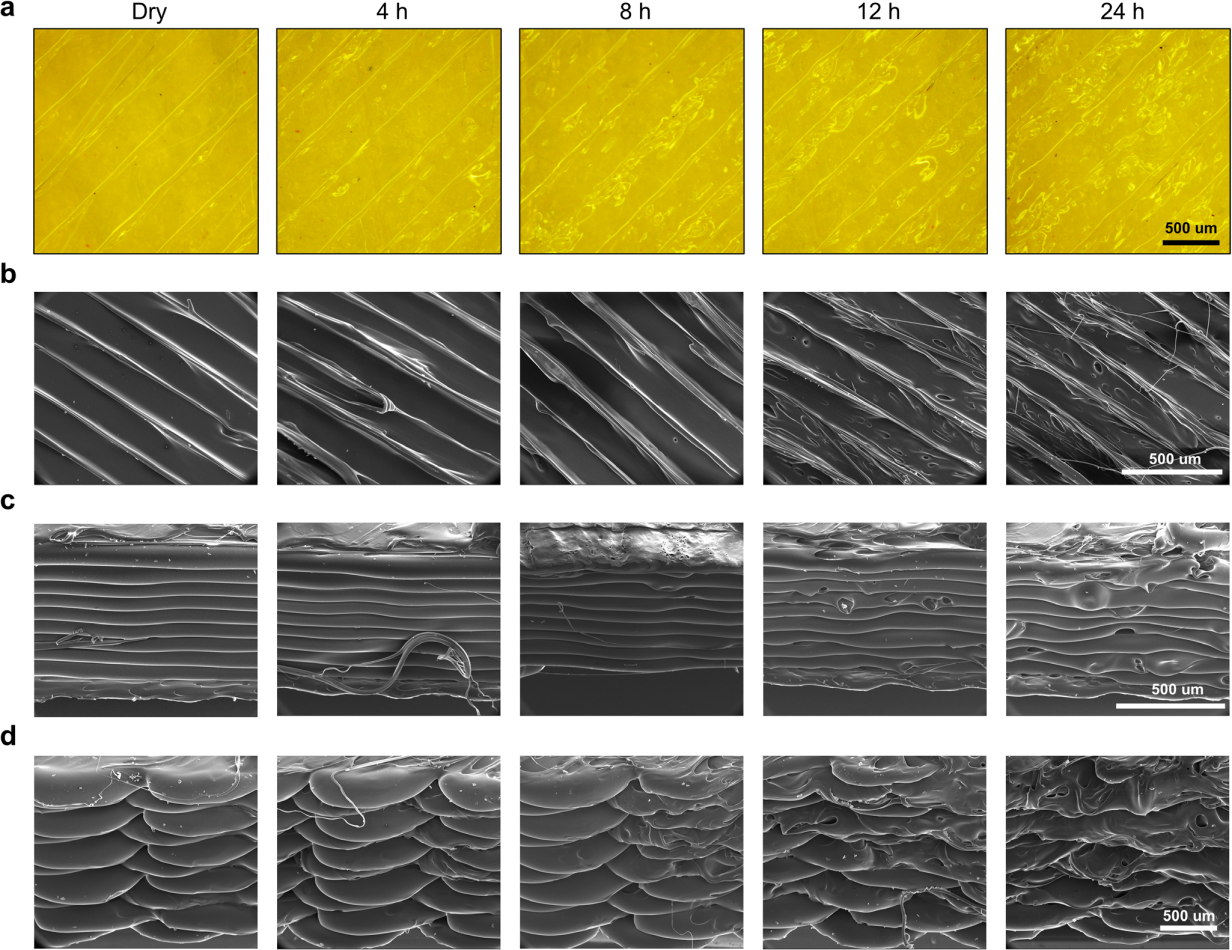
Microscopy images to understand the effect of filament immersion time on the morphology of 3D printed samples (scale bars = 500 µ*m*). (a) Digital optical microscopy images showing the surface morphology of printed specimens with different filament immersion times. (b‐d) SEM images of the surface morphology (b) and cross‐sectional views of outer wall (c) and interior (d) with different filament immersion times.

Microscopy analysis was performed to investigate the effect of moisture absorption on the structural integrity of the printed specimens. Optical microscopy images, presented in Figure 3a, reveal that while the dry specimen exhibited a smooth and uniform surface, the humidified samples displayed increasingly irregular morphologies and distinct voids as immersion time progressed. Scanning electron microscopy (SEM) was utilized to further examine the morphological characteristics of the printed specimens, specifically focusing on the surface, outer walls, and internal cross‐sections (Figure 3b–d). SEM observations revealed a clear immersion‐time‐dependent degradation. For 4‐h and 8‐h immersions, the uniformity of the printing path began to be disrupted, accompanied by the initial formation of discrete micropores. As immersion time extended to 12 and 24 h, these structural defects evolved into a porous topology with a significantly higher pore density. Highly magnified SEM images highlighting the structural defects are provided in Figure S3. The presence of these micropores compromises structural stiffness by reducing the effective load‐bearing area and disrupting the stress distribution continuity. This behavior aligns with theoretical homogenization frameworks for porous structures, such as the Gibson–Ashby model [27], where the accumulation of internal voids drives a macroscopic reduction in the Young's modulus of the structures. Specifically, the aggravation of structural imperfections with extended immersion periods provides a physical basis for the progressive reduction in Young's modulus of the printed part, observed as a function of immersion time. We note that, while the degradation of the filament itself is primarily attributed to plasticization, the mechanical performance of the printed parts is fundamentally governed by these structural imperfections. Thus, along with intrinsic material degradation, these architectural defects serve as a major mechanism driving the mechanical decline of the final components.

### 2.2 Diffusion Model‐Based Anomaly Detection Framework

**FIGURE 4:**
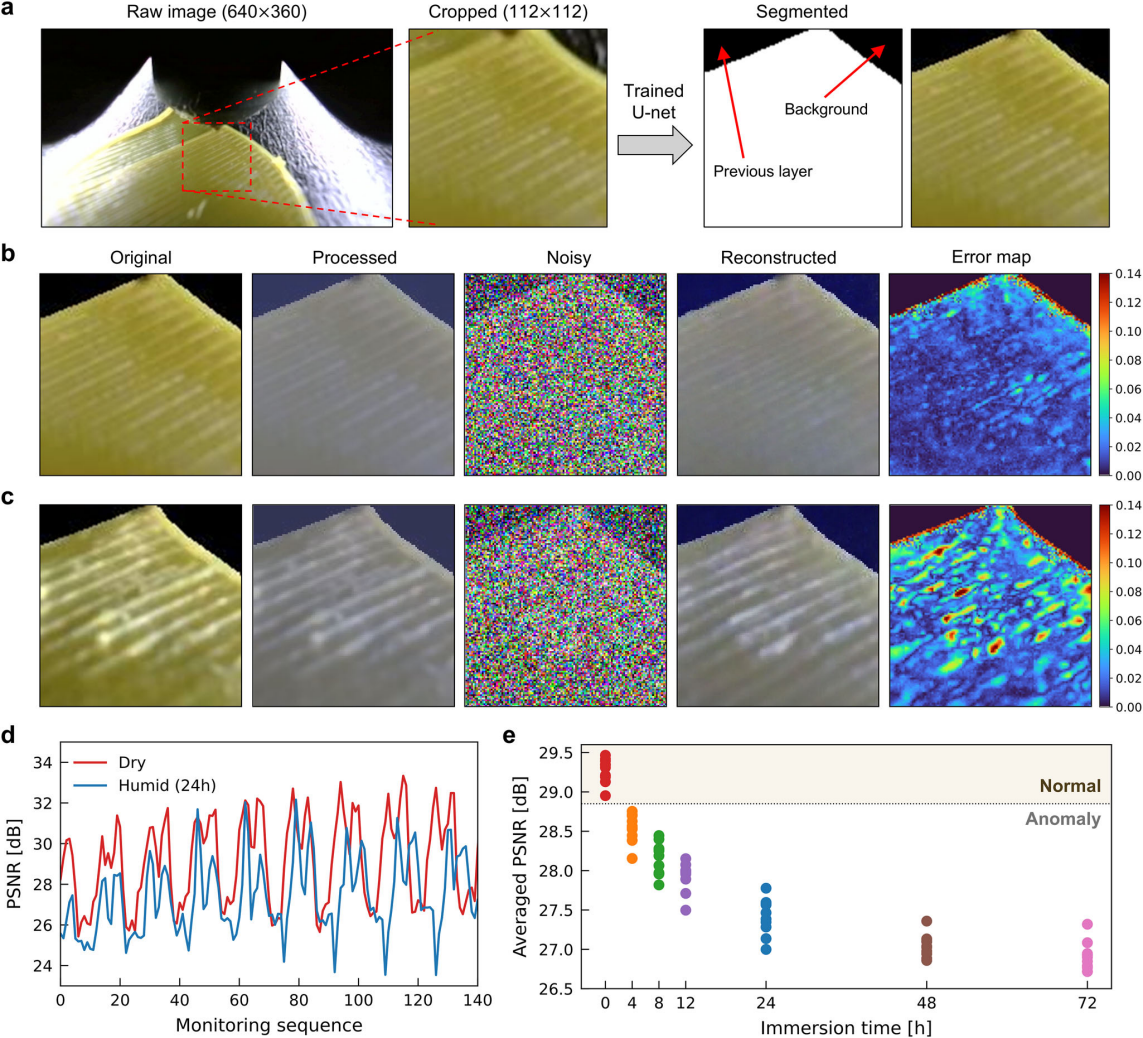
(a) Workflow of the image processing. The raw image acquired via the integrated camera is processed through cropping and image segmentation to isolate the region of interest. (b, c) Visualization of the image reconstruction process using the diffusion model. The sequential images present the original image, the preprocessed input, the addition of noise, the reconstructed image, and the resulting error map for dry (b) and humidified (c) conditions. (d) PSNR results of sequential images captured during printing a single specimen for each condition. (e) Averaged PSNR values across different filament immersion times (*n*  =  10).

Diffusion models provide a powerful framework for anomaly detection. Since these models are trained exclusively on normal data, they are effectively applicable even in scenarios with a severe imbalance between normal and anomalous samples. Furthermore, diffusion models do not require discrete labels, offering the advantage of continuously evaluating the deviation from the normal data through reconstructions. Building on these advantages, a diffusion model‐based anomaly detection is devised for real‐time monitoring of the FFF 3D printing process. A total of 161 images were acquired per specimen in situ during the printing process, without pausing or interrupting the print, using the monitoring setup in Figure 1b. It is of note that the initially printed layer was excluded from image acquisition because its thin and semi‐transparent nature makes it difficult to accurately characterize the surface features. As shown in Figure 4a, the collected images (640 × 360 pixels) were cropped to a size of 112 × 112 pixels around the printer nozzle tip, which is to focus exclusively on the surface characteristics recently deposited at the moment. Then, image segmentation was performed using a U‐Net segmentation model to eliminate the background and previous layers from the analysis. A U‐Net architecture with a pre‐trained ResNet34 backbone [28] was implemented and fine‐tuned for our specific 3D printing image dataset. The trained image segmentation model demonstrated excellent performance, achieving a mean IoU (Intersection over Union) of 0.9710 and a mean Dice score of 0.9849. The segmented images then undergo pre‐processing, including normalization and brightness adjustment, to serve as inputs for the diffusion model. We note that the diffusion model is trained exclusively on images of specimens printed with dry filaments to establish a baseline for normal printing conditions. The anomaly detection process involves a sequential noise injection (forward diffusion process) and subsequent reconstruction (reverse process). In the forward process, a processed image *x*_0_ is transformed into a diffused sample *x_t_* by introducing Gaussian noise according to a variance schedule β_*t*_ as follows:

(1) $$x_{t}=\sqrt{{\bar{\alpha }}_{t}}\;x_{0}+\sqrt{1-{\bar{\alpha }}_{t}}\varepsilon ,\varepsilon ∼N\left(0,I\right)$$

where

(1) $$\alpha_{t}=1-\beta_{t}\;\text{and}\;{\bar{\alpha }}_{t}=\prod_{i=1}^{t}\alpha_{i}$$

representing the cumulative product of noise scales. For the reverse process, the trained diffusion model iteratively removes noise to reconstruct the original data. This denoising step is modeled based on a Markov chain where the transition probability is defined as follows,

(2) $$p_{\theta }\left(x_{t-1}\left|x_{t}\right.\right)=N\left(x_{t-1};\mu_{\theta }\left(x_{t},t\right),\sigma_{t}^{2}I\right)$$

In detail, the sample at the previous timestep *x*_*t* − 1_ is recovered using the noise predicted by the model, ε_θ_(*x_t_*,*t*), as expressed as follows,

(3) $$x_{t-1}=\frac{1}{\sqrt{\alpha_{t}}}\;\left(x_{t}-\frac{1-\alpha_{t}}{\sqrt{1-\alpha_{t}}}\varepsilon_{\theta }\left(x_{t},t\right)\right)+\sigma_{t}z,\;\;z∼N\left(0,I\right)$$

Here, σ_*t*_ is random variation during the sampling process, ensuring the stochastic nature of the reconstruction. Detailed descriptions of the training processes for the image segmentation and diffusion models are provided in the Methods section. The average processing time, encompassing image segmentation, diffusion model‐based reconstruction, and PSNR calculations, was 176.9 ms per image.

Figure 4b,c present the original, processed, noisy, and reconstructed images, along with the corresponding error maps, for the dry and humidified filament cases, respectively. The results demonstrate that while the model achieves high reconstruction performance for dry conditions, as shown in Figure 4b, it exhibits significant reconstruction errors along the printing path for humidified specimens, as shown in Figure 4c. This discrepancy arises because the model, having learned only the features of dry specimens, fails to accurately reconstruct the irregular surface textures induced by moisture. The reconstruction results for different immersion times are presented in Figure S4, demonstrating that the reconstruction error increases progressively with immersion time. This reconstruction error serves as a key indicator of the deviation from the normal (dry) condition, effectively capturing the anomalous printing behavior induced by moisture absorption. In other words, a higher reconstruction performance, characterized by a lower reconstruction error, signifies a closer proximity to the normal condition. Conversely, a decline in reconstruction performance indicates a greater deviation from the dry state, reflecting the presence of moisture‐induced anomalies. To quantitatively evaluate the reconstruction performance of the diffusion model, the Peak Signal‐to‐Noise Ratio (PSNR) was utilized. PSNR is defined as the ratio between the maximum possible pixel value (MAX_I_) and the Mean Squared Error (MSE) of the reconstructed image relative to the original, expressed on a logarithmic decibel scale as follows:

(4) $$\mathrm{PSNR}\;=10\cdot \log_{10}\left(\frac{{\mathrm{MAX}}_{I}^{2}}{\mathrm{MSE}}\right)$$

Figure 4d presents the PSNR values for images collected during the printing of a single specimen under both dry and humidified (24‐h immersion) conditions. Although there is no distinct boundary between the PSNR values of the dry and humidified specimens, it is evident that the humidified specimens generally exhibit lower PSNR values. Furthermore, observation of the PSNR plots for various immersion times, as shown in Figure S5, reveals a consistent downward shift as the immersion duration increases. Consequently, comparing the average PSNR values of each specimen can represent a promising metric for anomaly detection. To verify the performance of the proposed model, TPU filaments were immersed in water for durations of 4, 8, 12, 24, 48, and 72 h; subsequently, ten specimens were printed for each immersion condition and dry condition to collect the test image dataset. The PSNR values were calculated for the dry condition and six humidified conditions from the reconstruction process of the proposed diffusion model, and the averaged PSNR values for each specimen are plotted against immersion time in Figure 4e. A clear distinction is observed between the dry and humidified conditions, as shown in dashed lines in Figure 4e, highlighting excellent anomaly detection performance. Furthermore, the PSNR values exhibit a consistent decline as immersion time increases. We note that although there were slight overlaps in PSNR values across different immersion times, the model demonstrated a clear correlation between immersion time and PSNR values. This trend is attributed to the fact that the model was trained exclusively on “dry” specimens. Consequently, increasing immersion time intensifies visual defects, resulting in progressively lower reconstruction performance. This indicates that the model is capable of not only anomaly detection but also real‐time assessment of the degree of moisture‐induced degradation in the part.

### 2.3 Real‐Time Monitoring System Evaluating the Degradation Level and Mechanical Properties

**FIGURE 5:**
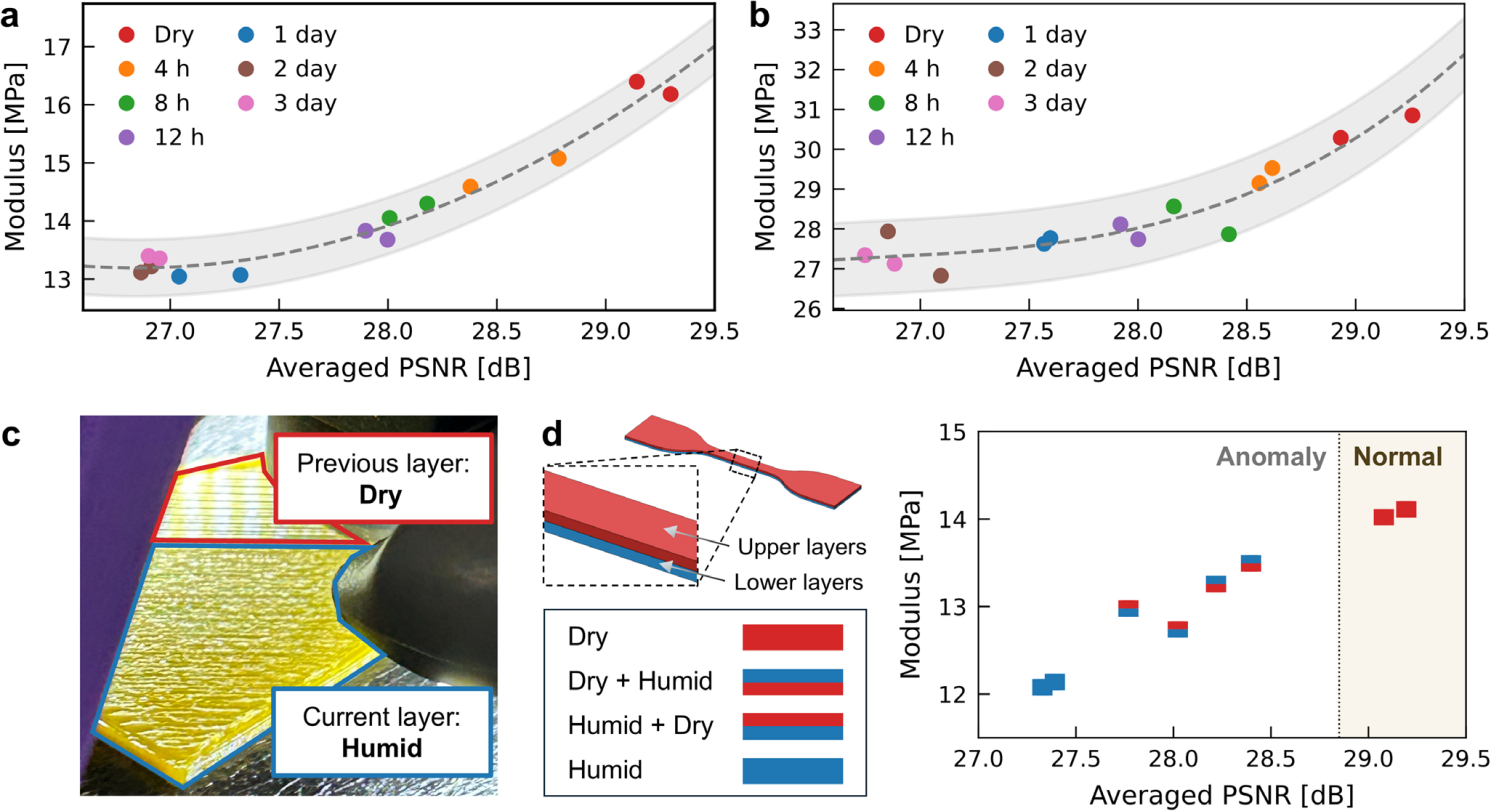
(a, b) Young's modulus as a function of averaged PSNR for as‐printed (a) and oven‐annealed (b) TPU specimens printed with filaments subjected to different moisture immersion times (*n*  =  2). (c) Image of a specimen during printing with filaments of combined moisture levels, showing the transition from dry to humidified filament. (d) Young's modulus vs. averaged PSNR plot for specimens printed using filaments with combined moisture levels (*n*  =  2).

Our framework successfully assessed the degree of moisture‐induced damage, represented by immersion time. This suggests that nondestructive evaluation of mechanical performance is achievable by establishing a robust correlation between the degree of moisture‐induced damage and mechanical properties. Specifically, as depicted in Figure 2g, a mechanical property map was constructed considering operational conditions (here, as‐printed and oven‐annealed) and the extent of moisture damage. Furthermore, Figure 4e illustrates the correlation between PSNR values and moisture‐induced damage. These correlations facilitate PSNR‐based evaluation of Young's modulus for both as‐printed and oven‐annealed states, as shown in Figure 5a,b. The dashed lines represent the trendlines, which show the positive correlation between PSNR and the mechanical properties. The shaded areas indicate an offset of ± 3% from the trendlines to account for experimental property variations in printed TPU parts. We note that the mechanical properties of TPU parts are highly sensitive to environmental factors, such as temperature and humidity. Therefore, a precise mechanical property map corresponding to specific operating conditions is essential. Under these considerations, our method enables real‐time, nondestructive evaluation of the mechanical performance of printed parts, leveraging the reconstruction performance of the diffusion model.

While the previous sections focused on parts printed using filaments with a uniform degradation, a more realistic scenario was investigated where the degree of degradation varies during the printing process. Four different filament conditions were examined: Dry, Dry+Humid, Humid+Dry, and Humid. For the Humid condition, filaments were immersed in water for 24 h prior to printing. Dry+Humid denotes the specimens where the first half was printed with dry filament and the second half with humid filament, and Humid+Dry refers to the reverse configuration. These transitions between different degradation levels were achieved by thermally fusing the filaments using a filament connector, as shown in Figure S6a. Figure 5c shows the surface characteristics of a Dry+Humid specimen at the midpoint of printing, where the smoothly printed previous layer is covered by a degraded and irregular layer due to the degradation transition. The correlation between PSNR and the mechanical properties for the above four cases was investigated in Figure 5d. It is noteworthy that the proposed model identified only the Dry case as normal, effectively detecting not only the fully humidified specimens but also those containing partially humidified segments as anomalies. In addition, the PSNR plots for the Dry+Humid and Humid+Dry specimens, shown in Figure S6b, show that the model could capture the transitions in degradation levels, from dry to humidified and vice versa, through abrupt shifts in PSNR values. In parallel, the Young's modulus of the Dry+Humid and Humid+Dry specimens exhibits intermediate mechanical properties between the Dry and Humid cases, consistent with the theoretical predictions of the Voigt model. The PSNR values obtained from the proposed model for the Dry+Humid and Humid+Dry also fall within the mid‐range, highlighting a strong correlation between PSNR metrics and mechanical properties. This capability of the proposed model is achieved by monitoring the entire printing process to calculate average PSNR values instead of monitoring a single point in time. Meanwhile, the Humid+Dry specimens exhibit slightly lower PSNR values and Young's modulus than those of the Dry+Humid specimens. This asymmetry is attributed to the persistence of the print quality degradation introduced for the Humid+Dry case. The transition from humidified to dry filament did not immediately recover print quality, as subsequent layers were deposited on a degraded surface, requiring several layers for full recovery. Interestingly, the proposed model could capture the asymmetry as well. In conclusion, these results validate the robustness of the proposed framework and underscore its capability for real‐time, in situ monitoring of both the extent of moisture‐induced degradation and the resulting mechanical properties.

**FIGURE 6:**
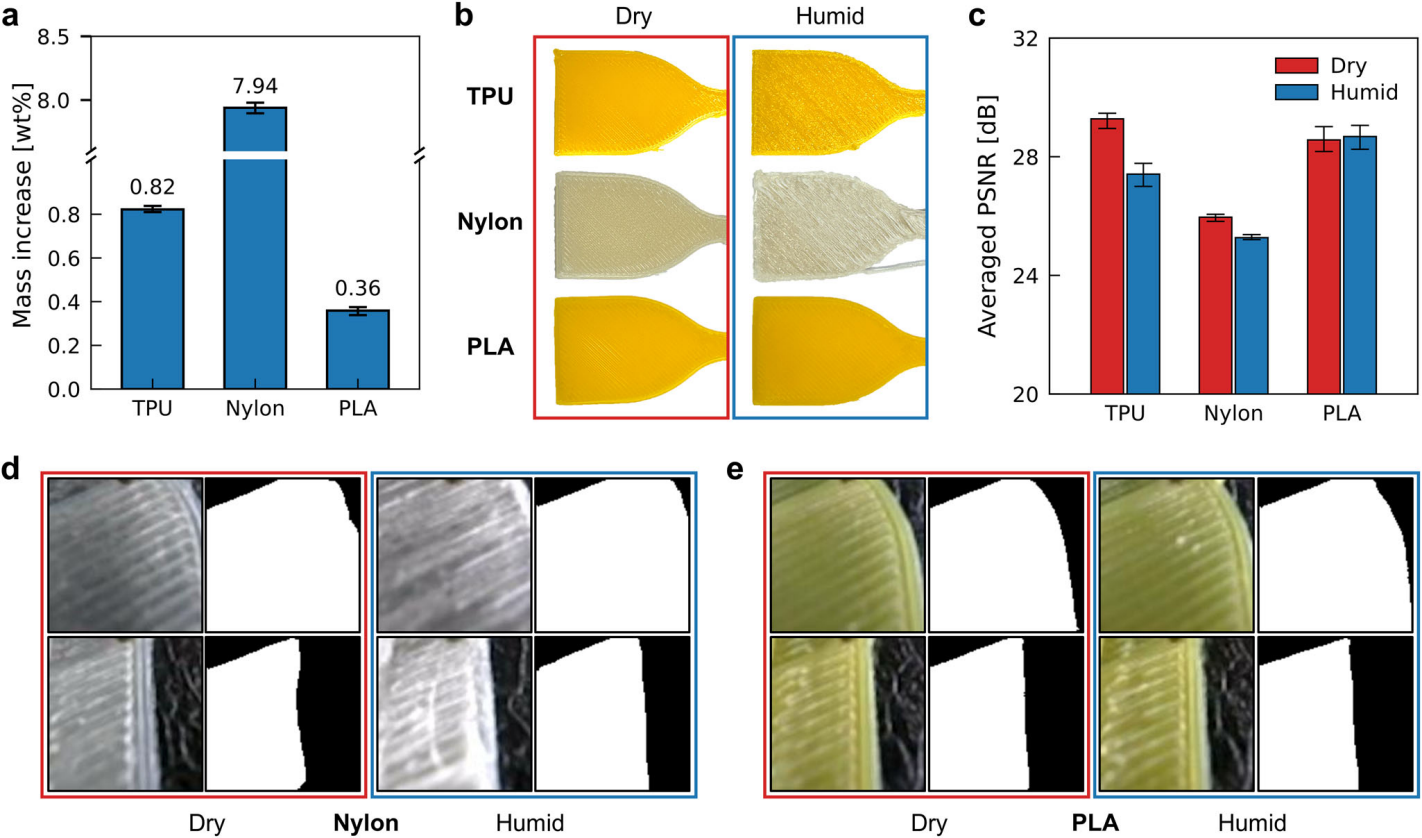
Generalization testing of the proposed framework across different filament materials. (a) Mass increase of TPU, Nylon, and PLA filaments after water immersion (*n*  =  3;  error bars indicate minimum and maximum values). (b) Surface images of printed parts using the three filament materials under dry and humid conditions. (c) Averaged PSNR values for the different filament materials under dry and humid conditions (*n*  =   3). (d, e) Images acquired via the monitoring system and corresponding segmentation masks for Nylon (d) and PLA (e) under dry and humid conditions.

To assess the generalization capability, we tested the proposed model on two additional filaments: PLA (yellow) and Nylon (translucent white). As shown in Figure 6a, the filaments exhibited varying degrees of mass increase after 24 h of immersion, indicating their distinct water uptake properties. Nylon exhibited substantial moisture absorption, approximately ten times that of TPU, whereas PLA showed relatively minimal water uptake, absorbing less than half the amount of water compared to TPU. Figure 6b presents the surface morphologies of the printed specimens for each filament and condition. It can be shown that Nylon displayed a more severe deterioration in printing quality under humidified conditions compared to TPU, whereas PLA maintained a smooth surface even after moisture exposure. The proposed model, trained solely on dry TPU specimens, was applied to different filament materials under dry and humidified (24‐h immersion) conditions, and the PSNR results are presented in Figure 6c. Remarkably, the model was capable of distinguishing between dry and humidified conditions for Nylon filaments, even though the model had seen the TPU printing process only. This result implies that the model effectively learned the morphologies of printing paths in “normal” conditions and highlights the potential generalizability of the proposed framework to diverse material types and colors of the filaments. The overall decrease in PSNR values of Nylon is attributed to the degraded segmentation performance on translucent white specimens, illustrated in Figure 6d, which is because the image segmentation model was trained exclusively on yellow specimens. The model's performance could be further improved by fine‐tuning the proposed framework or by training it from scratch using images from Nylon specimens. In contrast, PLA filaments exhibited no significant difference in PSNR between dry and humidified conditions (Figure 6c). This trend is consistent with images captured by the USB camera in our system, as shown in Figure 6e. The image segmentation model accurately identified the yellow specimens regardless of the material type; however, the lack of discernible visual differences between dry and humidified conditions of PLA led to a failure in distinguishing between the two, revealing the limitation of the proposed model. In summary, the proposed framework exhibits potential scalability across different material types and colors of the filaments in the condition that discernible visual differences are present under anomaly settings. To achieve this, segmentation and diffusion models need to be retrained or fine‐tuned according to the specific characteristics of the target application. Furthermore, it is worth noting that moisture‐induced degradation in mechanical performance can be attributed to either intrinsic material property degradation or microscopic structural defects. While the proposed model identifies anomalies based on visual discrepancies on the surface of the printed parts, it has a limitation in distinguishing the specific underlying source of degradation; addressing this would be an interesting line for future research.

## 3 Conclusions

This study successfully demonstrates a novel vision‐based real‐time monitoring system for FFF 3D printing, specifically devised to detect subtle moisture‐induced anomalies. Our investigation reveals that moisture absorption in TPU filaments leads to significantly degraded surface quality and up to about 20% reduction in mechanical performance compared to dry filaments. The microscopy images confirmed that the evaporation of absorbed moisture induced surface irregularities and internal micropores, which cause mechanical degradation in printed parts. To monitor these moisture‐induced defects, we have devised an AI‐driven in situ visual monitoring framework. Images captured during the printing process are preprocessed using a U‐Net‐based image segmentation model. A diffusion model is trained exclusively on images of parts printed with dry filaments (normal condition), and anomaly detection is performed using the PSNR as a reconstruction score metric. The model not only distinguishes between dry and humidified conditions but also successfully evaluates the extent of damage, where immersion time was employed as a metric to represent the damage. While those subtle differences in visual patterns among different degradation levels are identifiable through high‐resolution microscopy, they remain largely indistinguishable to conventional cameras or the human eye. The entire inference process, including image segmentation and reconstruction via the trained models, required only 176.9 ms per image, underscoring the rapid inference capability of the proposed framework. By leveraging the rapid inference and high sensitivity to subtle differences in AI‐driven frameworks, the proposed approach enables the real‐time and nondestructive detection of such degradation using only a cost‐effective USB camera. Moreover, by mapping the immersion time to the mechanical property measurements established through experiments, the system enables the nondestructive assessment of the final part's mechanical properties without physical testing. From a sustainability perspective, this work demonstrates how AI‐driven, real‐time monitoring can support more resource‐efficient and reliable manufacturing by enabling early, nondestructive detection of defects, which results in reducing material waste, energy consumption, and trial‐and‐error practices. Furthermore, the ability to evaluate moisture‐induced degradation in situ opens up the possibility for real‐time process parameter optimization to compensate for such effects by providing diagnostic information, which will be addressed in future studies. While this study validates the framework using TPU filaments in FFF printing, the results establish a foundational proof of concept for monitoring moisture‐induced defects. This data‐driven methodology holds potential for other highly hygroscopic materials and other FFF‐based printers through the required retraining or fine‐tuning of the segmentation and diffusion model with datasets tailored to the target application, in the condition that discernible visual differences are present under anomaly cases. Therefore, further research is required to determine the specific thresholds of discernible visual difference that the current setup can detect across various materials. Additionally, it would be interesting to investigate whether multi‐camera or multi‐angle imaging can capture the comprehensive visual information required to detect even more subtle visual anomalies. Lastly, future work should investigate the framework's generalizability to a broader range of manufacturing defects that manifest as discernible visual anomalies. Overall, we expect this research to enhance production quality and expand the industrial utility of 3D printing in the advanced manufacturing fields.

## 4 Methods

### 4.1 3D Printing

A Bambu Lab A1 mini 3D Printer was used for the printing. To enable real‐time monitoring, a commercial high‐speed USB camera module (ELP, China) and USB‐powered LED strip lights (PAUTIX, China) were integrated into the print head using a custom‐designed mount, as illustrated in Figure 1b. Images were captured during the printing process without pausing the printer head. A commercial TPU filament (1.75 mm, yellow, Overture, USA) was printed at a nozzle temperature of 240°C, a bed temperature of 30°C, and a printing speed of 35 mm/s. The specimens consisted of 10 layers with a 0.2 mm layer thickness. Specimens were printed with 100% infill density with a raster angle of 45° and 2 wall loops. The print direction was set from front to back in order to ensure clear visibility of newly printed regions, as shown in Movie S1. To investigate the effect of moisture on the filament, the TPU filaments were submerged in water at 17.5°C. This process was designed to accelerate the prolonged exposure of the filament to a humid environment. The surfaces of the immersed filaments were thoroughly wiped with paper towels before printing, ensuring that only the internal moisture content influenced the results without any potential interference from surface moisture. For the oven‐annealed specimens, printed samples were cured in an oven at 50°C for 4 h, followed by cooling at room temperature for 24 h prior to testing. PLA filament (1.75 mm, yellow, Overture, USA) was printed at a nozzle temperature of 210°C, a bed temperature of 70°C. Nylon filament (1.75 mm, translucent white, Creality, China) was printed at a nozzle temperature of 250°C, a bed temperature of 60°C. Other printing conditions were kept the same with the TPU filament.

### 4.2 Material Characterization

For characterizing the mechanical behavior, ASTM D412 Type C specimens were fabricated, and a ZwickiLine universal testing machine (UTM) (ZwickRoell, Germany), equipped with a 500 N load cell and 1 kN screw grips (ZwickRoell, Germany), was employed to perform quasi‐static tensile tests under a strain rate of 200 mm/min. The Young's modulus was calculated based on the initial slope of the stress‐strain curve over a strain range of 0–0.02. The mass increase test for the filaments was conducted by immersing a 1‐meter‐long segment in water. Optical microscopy was used to examine the printed surfaces at 400× magnification. SEM was used to examine the surface morphology and cross‐sections of printed samples. For surface imaging, single‐layer prints were mounted on SEM stubs using carbon tape. For cross‐sectional analysis, ASTM specimens were printed to half‐length and sectioned. All samples were sputter‐coated with a gold–palladium layer (10 mA) to a thickness of 151 Å (surface) and 200 Å (cross‐section) prior to imaging. The accelerating voltage was set to 20 kV.

### 4.3 Image Segmentation Model

A U‐Net architecture with a ResNet34 backbone was employed, utilizing an encoder initialized with ImageNet pre‐trained weights from the Segmentation Models PyTorch library [29]. A total of 1449 images from nine specimens were manually annotated using LabelMe, and the dataset was partitioned into training and validation sets at an 8:2 ratio. To comply with the standard input dimensions of the backbone, images were resized to 224 × 224 pixels and normalized, while random brightness and contrast adjustments were applied to mitigate overfitting. The training process utilized the Adam optimizer with a learning rate of 10^−4^ and Binary Cross‐Entropy with Logits loss, incorporating a learning rate scheduler with a 10‐epoch patience to identify the optimal parameters over 100 epochs. The trained image segmentation model was validated using 644 unseen test images derived from four specimens (two dry conditions and two under 24‐h immersion condition). For the test dataset, the model demonstrated superior performance, achieving a mean IoU of 0.9710 and a mean Dice score of 0.9849, where the metrics are defined as follows,

(5) $$\mathrm{IoU}\;=\frac{TP}{TP+FP+FN}\;$$

(6) $$\mathrm{Dice}\;\mathrm{score}\;=\frac{2TP}{2TP+FP+FN}\;$$

where TP, FP, and FN represent true positives, false positives, and false negatives, respectively. The histogram plots for each metric are presented in Figure S7. Finally, the trained model was used for instantaneous segmentation, where the resulting masks were integrated with the original images to isolate the region of interest by removing irrelevant background and previous layer information. To ensure data quality, images where the background exceeded 70% of the area were discarded, resulting in approximately 143 images per specimen for anomaly detection.

### 4.4 Diffusion Model

As illustrated in Figure 1c, a U‐Net‐based diffusion model was developed for the anomaly detection task. The encoder (down‐sampling path) processes 112 × 112 pixel images through four stages, with channel capacities increasing to 96, 192, 192, and 384 as the resolution is halved at each stage. Each stage incorporates residual blocks with SiLU activation and group normalization to ensure stable gradient flow, while self‐attention blocks are integrated at resolutions of 28 × 28, 14 × 14, and 7 × 7 to capture long‐range spatial dependencies. The bottleneck layer further employs residual blocks coupled with an attention mechanism, and the decoder (up‐sampling path) mirrors the encoder's architecture by utilizing transposed convolutions and skip connections to recover high‐resolution features. Temporal dynamics are incorporated via 384‐dimensional sinusoidal positional embeddings injected into each residual block. For model training, a dataset comprising 5753 images from 40 specimens was used for training and 1152 images from 8 specimens for validation, all captured under dry conditions. A Denoising Diffusion Probabilistic Model (DDPM) was implemented with a cosine‐based variance schedule across T = 1000 diffusion steps, employing a mask‐based loss weighting strategy to disregard background errors. The network was optimized using the Adam optimizer (learning rate: 10^−4^, weight decay: 0.01) and an Exponential Moving Average (EMA, decay: 0.9999) over 800 epochs with a batch size of 64, while mixed‐precision training and gradient clipping ensured robust convergence. For efficient inference and image reconstruction, the Denoising Diffusion Implicit Model (DDIM) was adopted to skip 20 steps at a time, allowing high‐quality reconstruction in fewer iterations. The process begins by adding Gaussian noise to the input images corresponding to 50% of the total diffusion steps (T = 500), after which the pre‐trained U‐Net iteratively removes noise to recover the clean image. Finally, PSNR was calculated to evaluate reconstruction performance.

### 4.5 Inference Efficiency

The average inference time per image on an NVIDIA RTX A5000 GPU was 176.9 ms, which includes 6.39 ms for image segmentation and 170.5 ms for the subsequent steps involving noise injection, reconstruction, and PSNR calculation. This corresponds to a maximum inference rate of 5.65 Hz under the current setup. In this study, images were acquired per specimen at an image acquisition frequency of 0.1362 Hz; however, this acquisition frequency can be scaled up to the maximum inference rate if required.

## Author Contributions

**Jiyoung Jung**: investigation, writing – original draft, methodology, visualization, formal analysis. **Yuna Yoo**: investigation, writing – original draft, methodology, visualization, formal analysis. **Dharneedar Ravichandran**: methodology, visualization, writing – review and editing. **Dahyun Daniel Lim**: methodology, visualization, writing – review and editing. **Grace X. Gu**: conceptualization, investigation, formal analysis, writing – review and editing.

## Conflicts of Interest

The authors declare no conflicts of interest

## Supporting information




**Supporting File 1**: advs76062‐sup‐0001‐SuppMat.pdf.

**Supporting File 2**: advs76062‐sup‐0002‐Movie_S1.mp4.

## Data Availability

The data that support the findings of this study are available within the article and its supplementary material.
