# Supplementary material for: Learning Moisture‐Induced Damage From Vision: Diffusion Models for Real‐Time Monitoring of Additive Manufacturing Processes
Source: Adv Sci (Weinh). 2026 Jul 1:e76062. Online ahead of print. doi: 10.1002/advs.76062 (PMC13336440; doi:10.1002/advs.76062)
Supplement: Supplementary file 1 — Supporting File 1: advs76062‐sup‐0001‐SuppMat.pdf. [file ADVS-9999-e76062-s001.pdf]

Supporting Information

Learning moisture-induced damage from vision: Diffusion models for real-time monitoring of additive manufacturing processes

Jiyoung Jung[1,†], Yuna Yoo[1,†], Dharneedar Ravichandran[1], Dahyun Daniel Lim[1,2], and Grace X. Gu[1,*]

[1]Department of Mechanical Engineering, University of California, Berkeley, CA 94720, USA.
[2]School of Mechanical Engineering, Korea University, Seoul, Republic of Korea.
[†]These authors contributed equally to this work.
[*]Corresponding author: ggu@berkeley.edu

Supporting figures

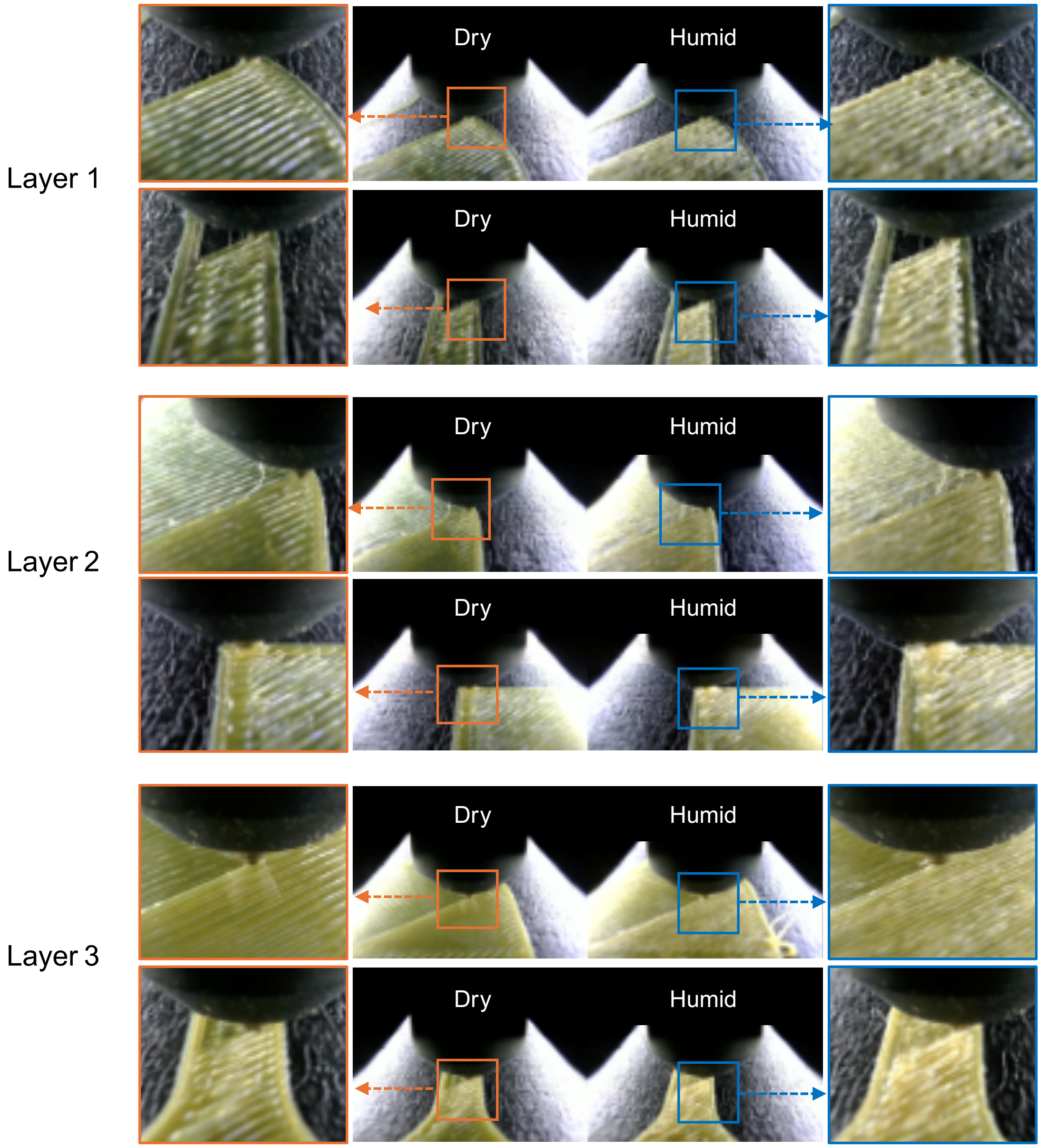

Figure S1. Representative key frames from layers 1, 2, and 3 under dry and humid conditions. Comparison of images captured at the same locations reveals surface degradation induced by moisture absorption in the filament.

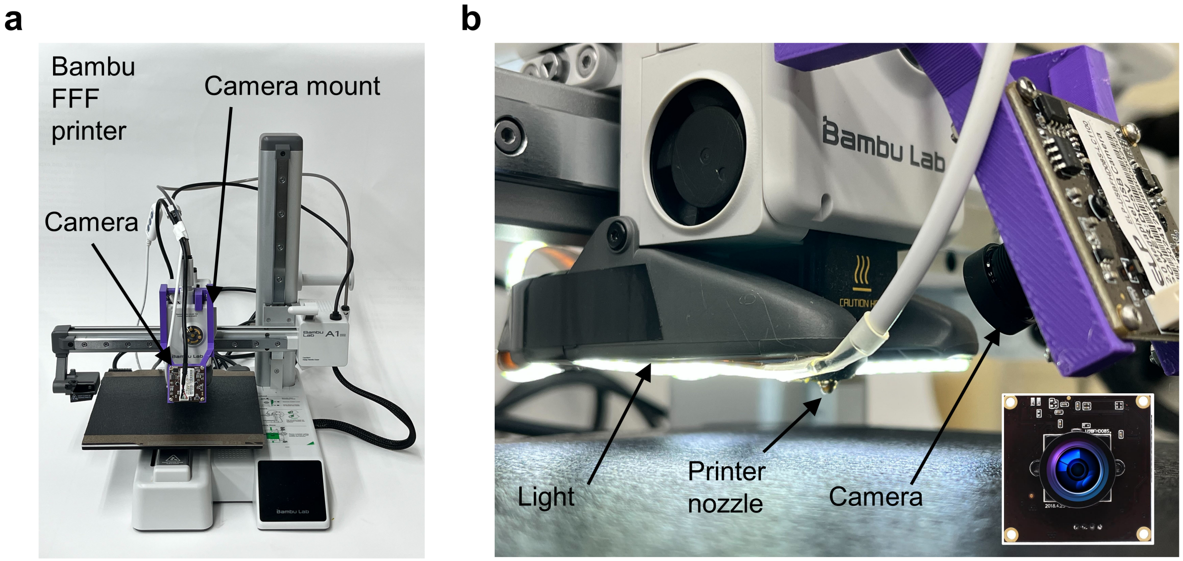

Figure S2. Experimental setup for a real-time, in-situ visual monitoring system. (a) Digital photograph of the overall monitoring setup and (b) magnified view of the monitoring system. A commercial optical USB camera is secured to the print head of a Bambu Lab A1 mini printer using a custom-designed camera mount. The camera is aligned to focus on the printer nozzle tip. To ensure visibility, an LED strip light is installed beneath the cooling fan, providing localized illumination.

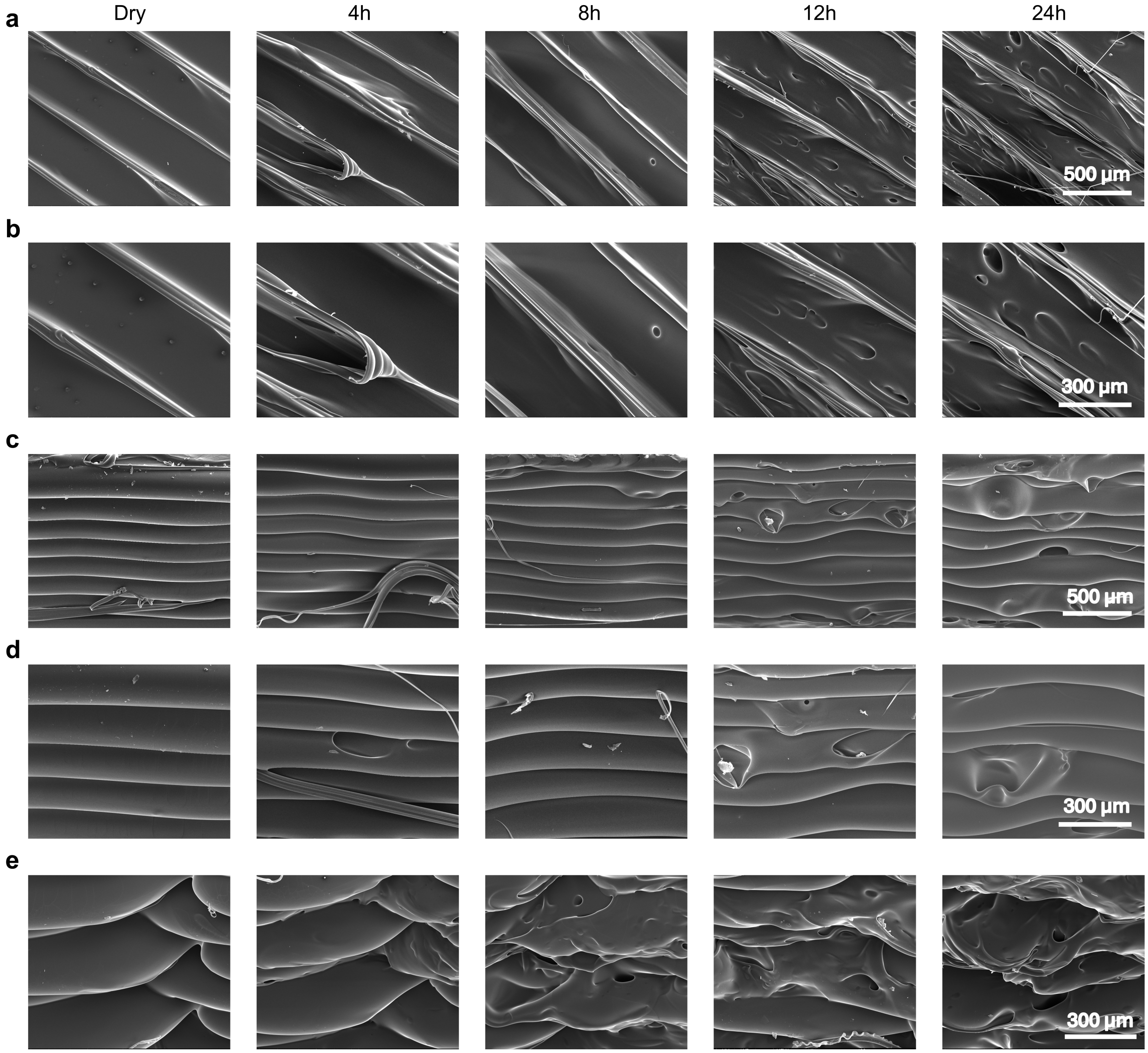

Figure S3. SEM images of the (a, b) surface morphology, (c, d) cross-sectional views of outer wall, and (e) cross-sectional views of interior for the printed specimens with different filament immersion times.

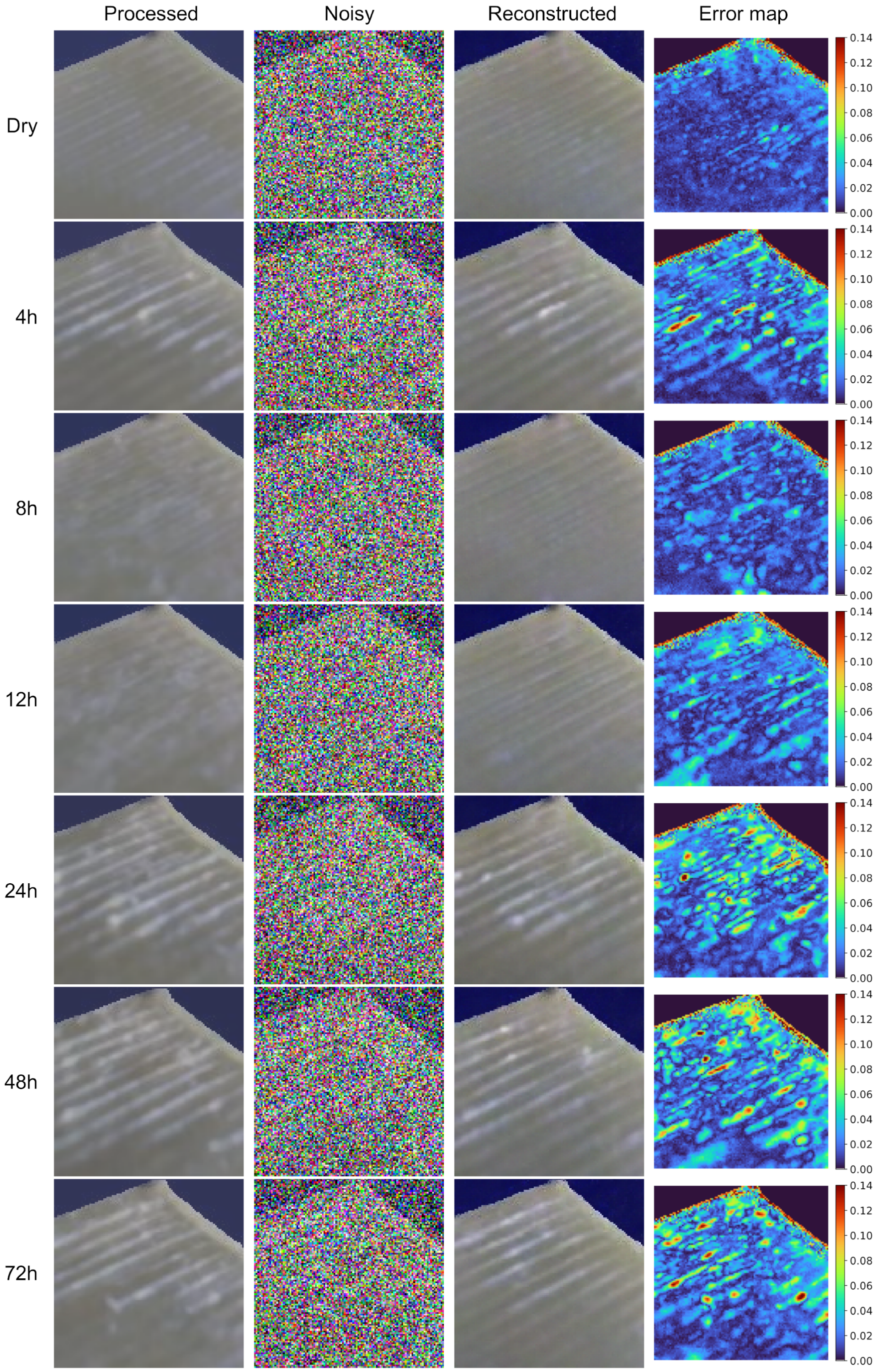

Figure S4. Visualization of the image reconstruction process using the diffusion model for dry and different humidified conditions. The sequential images present the preprocessed input, the addition of noise, the reconstructed image, and the resulting error map. The error maps reveal that the reconstruction error increases proportionally with the immersion time.

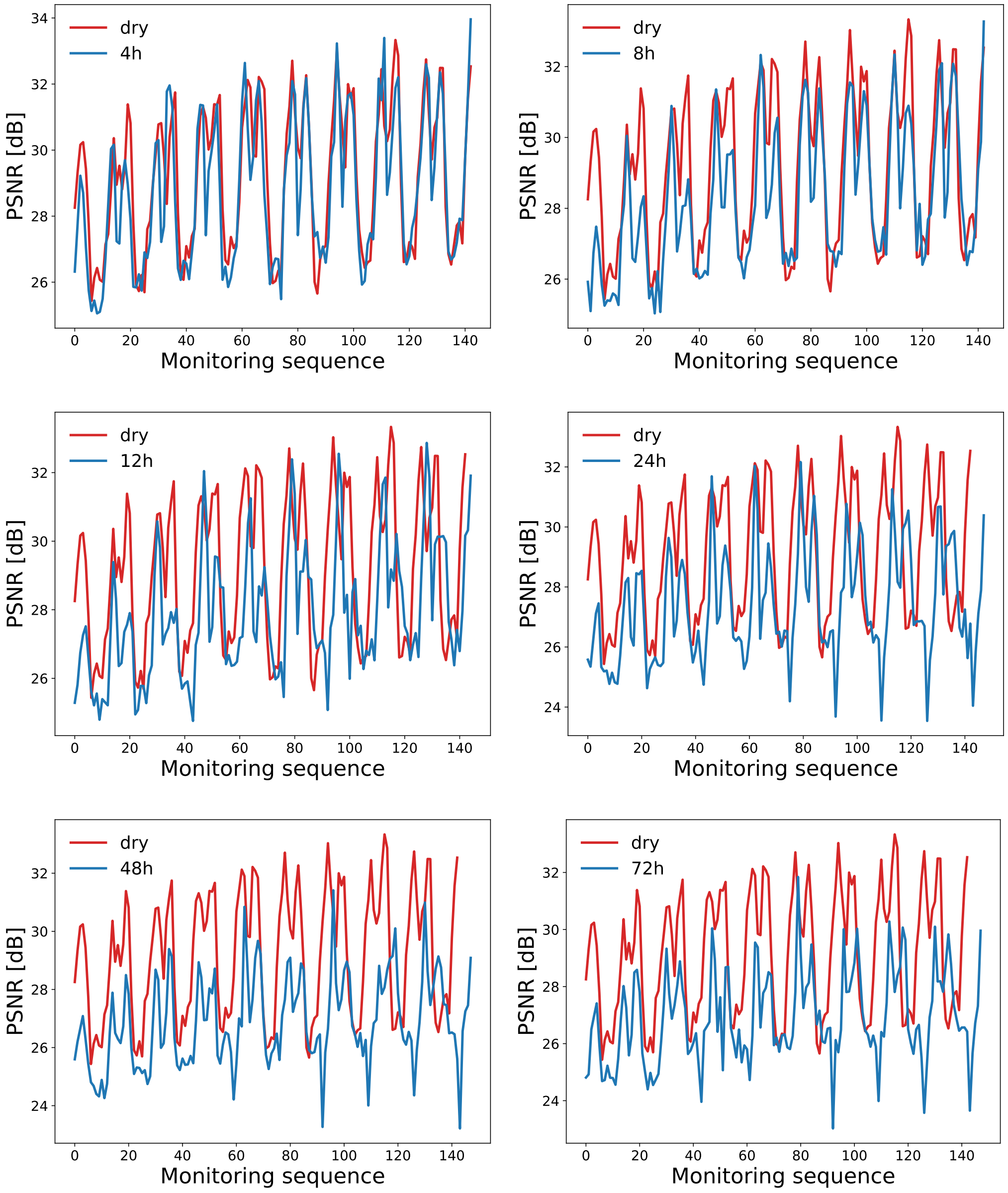

Figure S5. PSNR results of sequential images captured during printing the single specimen for dry and different humidified conditions. The results demonstrate a downward shift in the PSNR plots as the immersion time increases.

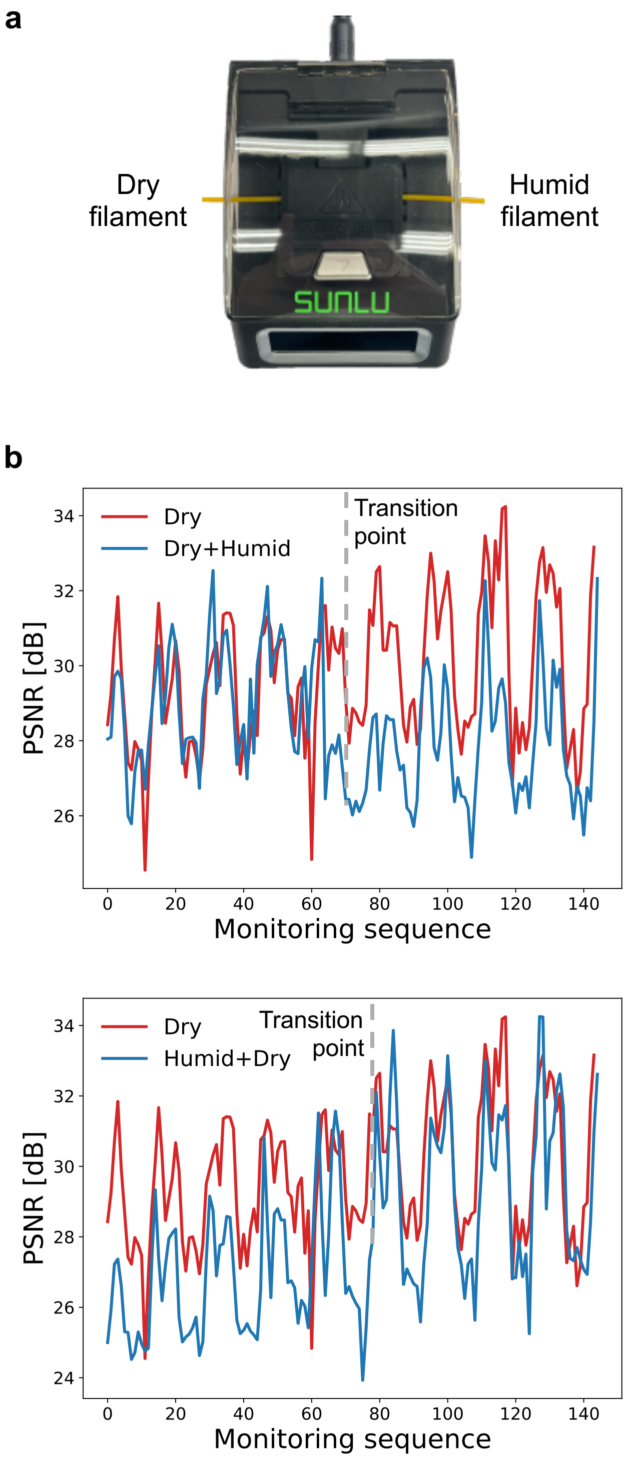

Figure S6. (a) Digital photograph of a filament connector utilizing thermal fusion for filament connecting. (b) PSNR results of sequential images captured during printing the single specimen for dry, Dry+Humid, and Humid+Dry. The transitions in degradation levels, from dry to humidified and humidified to dry, are captured through abrupt shifts in PSNR values.

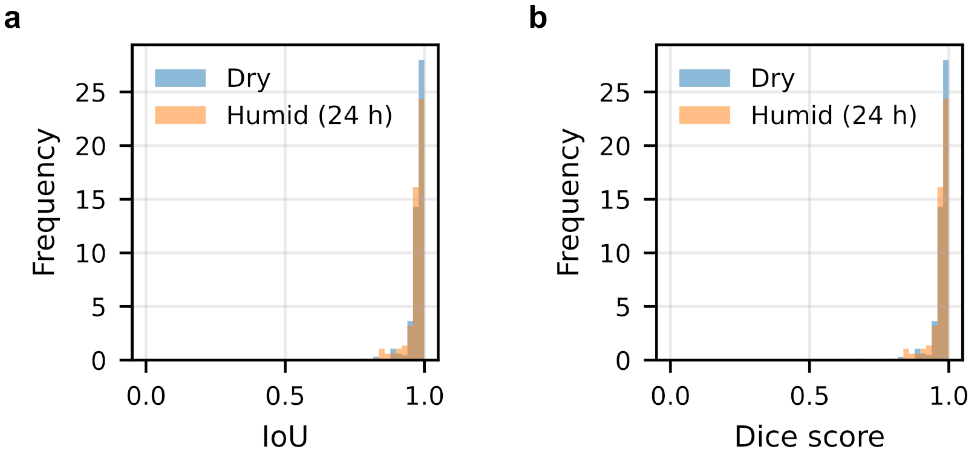

Figure S7. Performance evaluation of the image segmentation model on 664 unseen test images: (a) IoU and (b) Dice score. The test set includes images from specimens printed with dry and 24 h immersion filaments (two specimens each). The model, trained on both dry and humid conditions, demonstrates high segmentation accuracy with a mean IoU of 0.9710 and a mean Dice score of 0.9849.
